# Evapotranspiration Everywhere, All the Time: Towards a Unified View From Earth Observation

**DOI:** 10.1111/gcb.70898

**Published:** 2026-05-10

**Authors:** Joshua B. Fisher, Martha C. Anderson, Diego G. Miralles, Kanishka Mallick, Paul C. Stoy, Youngryel Ryu, Wim G. M. Bastiaanssen

**Affiliations:** ^1^ Schmid College of Science and Technology, Chapman University Orange California USA; ^2^ Hydrosat, Inc. Washington DC USA; ^3^ USDA‐ARS Hydrology and Remote Sensing Laboratory Beltsville Maryland USA; ^4^ Hydro‐Climate Extremes Lab (H‐CEL) Ghent University Ghent Belgium; ^5^ CESBIO University of Toulouse, CNRS, CNES, INRAE, IRD Toulouse France; ^6^ Department of Biological Systems Engineering University of Wisconsin – Madison Madison Wisconsin USA; ^7^ Department of Landscape Architecture and Rural Systems Engineering Seoul National University Seoul South Korea; ^8^ Department of Water Resources Management, Faculty of Civil Engineering Delft University of Technology Delft the Netherlands

**Keywords:** ECOSTRESS, evapotranspiration, Hydrosat, Landsat, MODIS, remote sensing, thermal infrared, VIIRS

## Abstract

Scientists want to know everything, everywhere, and all the time. This is particularly true in Earth science, where we seek to understand processes that span from the molecular to the planetary scale in how the world works, how it affects us, and how we impact it—especially the water cycle. Evapotranspiration (ET) was the last component to be measured in closing the water cycle: for decades, closing the water budget meant adding up all the measurable components, then inferring ET as the residual. Early measurements relied on water loss from pans and weighing lysimeters, followed by sensors inserted into plants to monitor sap flow and leaf chambers capturing transpiration. Scaling up to ecosystems became possible through eddy‐covariance flux towers and further across landscapes through proximal sensing with drones, aircraft, and, ultimately, with satellites. While enormous progress has been made to measure or estimate ET everywhere and all the time, no single approach has yet achieved both simultaneously. Flux towers help with all the time, but not everywhere. Satellites can do everywhere, but not all the time (except, in part, for geostationary satellites, though with insufficient spatial coverage and resolution). A new advent of smallsat constellations is moving us to everywhere and all the time in detail, though we are only in the beginning of that era. This paper discusses the evolution and revolution of Earth observation for ET, as we advanced from the first Landsat and development of ET models through the progression of increasingly higher spatiotemporal resolution across international space agencies and commercial industry with increasing ET model sophistication, cloud computing, and machine learning. We continue to march ahead towards ET everywhere, all the time, and use that knowledge to better manage water and sustain our planet.

## Graphical abstract

This paper discusses the evolution and revolution of Earth observation for evapotranspiration (ET), as we advanced from the first Landsat satellite and development of ET models through the progression of increasingly higher resolution across international space agencies and commercial industry with increasing ET model sophistication, cloud computing, and machine learning.

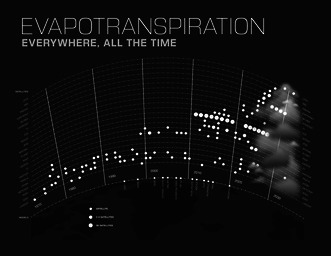

## 1 In the Beginning

**FIGURE 1:**
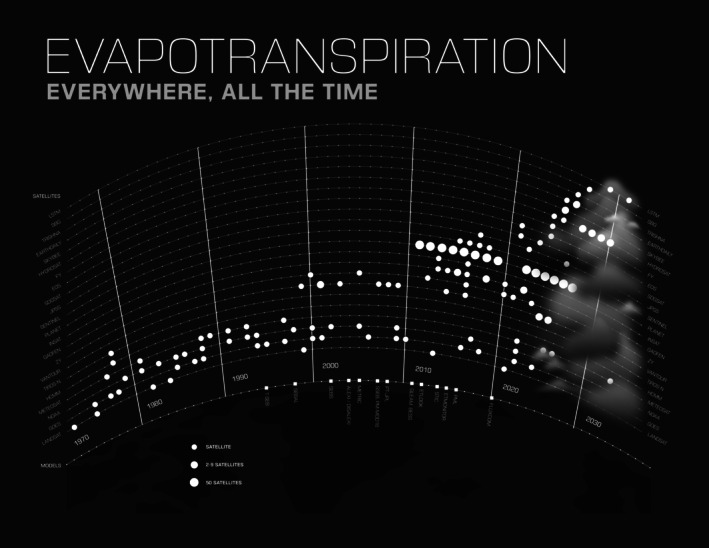
The trajectory of evapotranspiration from Earth Observation. Satellites and models have advanced in spatiotemporal resolution and sophistication, marching us towards evapotranspiration everywhere, all the time for increasing science and applications.

In the beginning (in 1972), the United States (US) National Aeronautics and Space Administration (NASA) created Landsat‐1 (Wulder et al. 2019) (Figure 1). The multi‐spectral scanner (MSS) on Landsat‐1 was able to see every part of the world in great detail (60 m). But frequent global revisits were not possible with a single satellite and no suitable helper was found; so, the US National Oceanic and Atmospheric Administration (NOAA) created the Geostationary Operational Environmental Satellite (GOES‐1) with the Visible and Infrared Spin Scan Radiometer (VISSR) (in 1975) (Menzel and Purdom 1994) and the Television Infrared Observation Satellite (TIROS‐N) with the Advanced Very High Resolution Radiometer (AVHRR‐1) (in 1978), among other instruments (Cracknell 1997). Also in 1978, NASA launched the experimental Heat Capacity Mapping Mission (HCMM) (Short and Stuart 1982). Meanwhile, the European Space Agency (ESA) launched the first geostationary Meteosat satellite (Meteosat‐1) with the Meteosat Visible and Infrared Imager (MVIRI) in 1977 (Legrand et al. 2001). These satellites prospered and the programs were fruitful with follow‐on satellites for many decades, knowing both high detail and regional‐to‐global coverage.

They were not the first satellite instruments ever, but they were the first to observe Earth in both visible and near‐infrared (VNIR) wavelengths, providing an indicator of vegetation health and activity through the calculation of the normalized difference vegetation index (NDVI) and related indices (Tucker 1979). NDVI alone is often a good predictor of ET, although it can fail under conditions of vegetation stress, varying weather conditions, thick canopies, or high soil evaporation (Joiner et al. 2018). Consequently, researchers compiled and combined weather data with NDVI to model ET with higher fidelity than with NDVI alone (Nemani and Running 1989).

HCMM, GOES‐1's VISSR, and Meteosat's MVIRI also contained another measurement wavelength: thermal infrared (TIR). TIR enabled direct detection of land surface temperature, which is related to how much water plants use (i.e., transpiration) via the cooling effect of evaporation (Fisher et al. 2017). In the same year (1978) that NOAA launched AVHRR‐1, NASA launched Landsat‐3 also with TIR (though the band failed shortly after launch). AVHRR‐2 followed closely behind with TIR capabilities in 1981. Landsat‐4 launched the next year in 1982 with an improved thermal band in the Thematic Mapper (TM) instrument (Anuta et al. 1984). All subsequent Landsat‐5‐9 missions have had TIR capabilities; likewise, all subsequent NOAA‐6‐19 missions have had TIR capabilities in their AVHRR instruments, and all subsequent GOES‐1‐19 and Meteosat‐1‐12 missions have had TIR capabilities.

## 2 The First Satellite‐Based ET Models

These first satellites providing VNIR and TIR data in the early 1980s enabled calculations of ET, with pioneering studies emerging a few years later into the late 1980s and early 1990s. Still, the development of models that used these types of data was formed prior to the satellites by using similar ground‐based instruments (e.g., Bastiaanssen and Roebeling 1993; Choudhury 1989; Jackson et al. 1977; Klaassen and van den Berg 1985; Menenti 1984; Moran and Jackson 1991; Nemani and Running 1989; Rosema et al. 1978; Schmugge and Becker 1991; Seguin and Itier 1983; Sellers 1985; Soer 1980; Taconet et al. 1986). These early models evolved through the late 1990s into some of the fundamental satellite‐based ET modeling frameworks still used today.

Emerging from this early satellite‐based model development was the Two‐Source Energy Balance (TSEB) model (Anderson et al. 2024; Norman et al. 1995). TSEB partitioned observed land surface temperature between soil and canopy, deriving separate energy fluxes between these components. TSEB became very useful for agricultural applications wherein crop water use is separated from evaporation from the inter‐row space. TSEB is still used today in the ALEXI/DisALEXI model developed by the US Department of Agriculture—Agricultural Research Service (Anderson et al. 2004), and in the ESA Sen‐ET platform (Guzinski et al. 2020).

Among the most influential of satellite‐based ET models was the Surface Energy Balance Algorithm for Land (SEBAL) model (Bastiaanssen et al. 1998). SEBAL applied the concept of “hot” and “cold” pixels to scale sensible heat flux within a given image, thereby avoiding explicit retrieval of aerodynamic properties. It was self‐calibrated without relying on ground data and was relatively simple to use. As such, it was adopted early by many users, and freely‐available code and accuracy gave it lasting power. SEBAL is still widely used today, e.g., as part of the OpenET system (i.e., geeSEBAL; Laipelt et al. 2021; Melton et al. 2022), and as part of Hydrosat's IrriWatch system used for agricultural management (Ghorbanpour et al. 2025; Tabatabaii and Dehghanisanij 2024).

Although TSEB and SEBAL emerged independently at about the same time, TSEB explicitly models the surface–atmosphere coupling, while SEBAL adopts a more empirical simplification. Both benefited from the relatively high spatial resolution of TIR data provided by the Landsat satellites (< 120 m) to quantify water use closer to the scale of management on the ground. In contrast, the 1–4 km TIR resolution of AVHRR and GOES tends to mix signals from multiple land cover types and management practices, limiting their utility for field‐scale applications.

## 3 The MODIS Era

The launch of the MODerate resolution Imaging Spectroradiometer (MODIS) instruments on board the Terra and Aqua satellites in the early 2000s, as part of NASA's Earth Observing System, marked a rapid expansion in satellite‐based ET data and models (Justice et al. 1998). MODIS provided VNIR and TIR data at 1 km or finer spatial resolution, daily, and global. Of additional note was that the Advanced Spaceborne Thermal Emission and Reflection Radiometer (ASTER) on Terra deployed an expanded 5‐band thermal imager at 90 m resolution, though this large amount of data meant that NASA was limited in their ability to downlink and store global spatial and temporal coverage (Abrams 2000). MODIS was also directly linked with the emergence of FLUXNET, which significantly increased the ability of modelers to validate their ET estimates at the scale of 1 km pixels across ecosystems using a wealth of high‐quality and consistent measurements from eddy covariance (Baldocchi et al. 2001).

As MODIS data began to accumulate, the Landsat program (managed by the US Geological Survey; USGS) was already on its fifth satellite (Landsat‐5, still operational at the time since launch in 1984) and sixth satellite (Landsat‐7, newly launched in 1999; Landsat‐6 crashed at launch), now having two decades of data across all its satellites. Likewise, at the turn of the millennium, AVHRR was on its ninth and tenth iterations on NOAA‐14 and NOAA‐15, and GOES was on its tenth satellite, both programs with many more missions to come. Some of the most widely used satellite‐based ET models were developed in the mid‐2000s based on Landsat and AVHRR data, and they were soon applied to the newly emerging MODIS data.

Around this time, in the mid‐2000s, six key satellite‐based ET models emerged, four originally designed primarily for local to regional scale applications and two for global scale applications, the former being the Surface Energy Balance System (SEBS) (Su 2002), Atmosphere‐Land Exchange Inverse disaggregation (ALEXI/DisALEXI) (Anderson et al. 2004), Mapping Evapotranspiration with Internalized Calibration (METRIC) (Allen et al. 2005), and Simplified Surface Energy Balance (SSEB) (Senay et al. 2007), and the latter being Priestley‐Taylor Jet Propulsion Laboratory (PT‐JPL) (Fisher et al. 2008) and Penman‐Monteith for MODIS product 16 (PM‐MOD16) (Mu et al. 2007).

All the local/regional‐scale models solved for the sensible heat flux and calculated ET as the residual of the energy balance. ALEXI/DisALEXI evolved from the TSEB model and was among the first to use the geostationary TIR data from GOES, primarily as a time differential to solve the energy balance for daily ET, then downscaled using Landsat or MODIS TIR. ALEXI/DisALEXI has been used for multi‐scale monitoring of water use, plant stress, and agricultural drought. METRIC and SSEB, which were derived from the SEBAL model, relied principally on Landsat TIR data and began to be widely used in agricultural and water resource management applications throughout the western US and other water‐limited regions.

PT‐JPL represented one of the first global‐scale satellite‐based ET models, leveraging the record of AVHRR as well as the climatological dataset compiled in the International Satellite Land‐Surface Climatology Project, Initiative II (ISLSCP‐II; Los et al. 2000). Unlike the energy balance approaches, which solved for sensible heat flux, PT‐JPL solved ET directly by downregulating the Priestley and Taylor (1972) potential ET model by using ecophysiological constraints that leverage a combination of VNIR, TIR, and weather sensitivities. Similarly, the PM‐MOD16 model was developed to solve ET directly using the Penman‐Monteith (1965) equation with adaptations for retrieving the resistances using globally available data. PM‐MOD16 became the operational ET model for the MODIS dataset, producing an enormous wealth of global data over the MODIS record.

Many of these models have since evolved beyond their original design scales. Models that originally had local foci have been adapted for global mapping, while models that were focused on global evaluations have now been adapted for field‐scale applications. SEBS was extended globally in one of the first unified ET intercomparisons, along with PT‐JPL and an interpretation of PM‐MOD16 (McCabe et al. 2013; Miralles et al. 2016; Vinukollu et al. 2011). ALEXI, which uses the time differential from geostationary satellites, can now use day/night temperature differences from polar orbiters like MODIS (Hain and Anderson 2017). Hot/cold pixel end‐member selections for SEBAL, METRIC, and SSEB, which were previously done manually, have now become automated (e.g., geeSEBAL, eeMETRIC, SSEBop) (Kilic et al. 2021; Laipelt et al. 2021; Senay et al. 2020).

## 4 VIIRS, Sentinel, ECOSTRESS, and the Next Generation of ET Models

About a decade after the MODIS instruments launched, NASA and NOAA partnered to launch the Visible Infrared Imaging Radiometer Suite (VIIRS) instruments on Suomi National Polar‐orbiting Partnership (Suomi NPP) in 2011, followed by NOAA‐20 in 2017 and NOAA‐21 in 2022, primarily to ensure continuity with MODIS (Endsley et al. 2025; Hillger et al. 2013). The VIIRS I5 band provided higher spatial resolution than MODIS at 375 m for VNIR and TIR, and it was able to provide the same temporal resolution (twice daily) with just one sensor due to its increased swath size. However, the number of spectral bands decreased from 36 with MODIS to 22 with VIIRS, making continuity imperfect especially for those products that used the missing MODIS bands.

A few years later in 2015, the ESA Copernicus program launched Sentinel‐2A for optical imaging (following Sentinel‐1A in 2014 for radar imaging). Although Sentinel‐2A did not have thermal bands, it did provide high‐resolution VNIR at 10 m (and shortwave infrared, SWIR, at 20 m) every 10 days. With the launch of Sentinel‐2B 2 years later in 2017, the revisit time shortened to 5 days (Li and Roy 2017). It was not until Sentinel‐3AB launched in 2016 and 2018 that the Copernicus program included TIR with the Sea and Land Surface Temperature Radiometer (SLSTR), though its resolution was coarser (1 km) (Yang et al. 2020).

At around this same time in 2016, geostationary capabilities were improved in the GOES‐R era with the launch of GOES‐16 with its Advanced Baseline Imager (ABI), followed by GOES‐17‐19 (Schmit et al. 2005). GOES‐16 improved on the previous GOES generation with four times the spatial resolution (0.5–2 km) and more than three times the number of spectral bands (i.e., 16). NOAA's National Environmental Satellite, Data and Information Service (NESDIS) currently uses these data to produce ET and drought (GET‐D) products (Fang et al. 2019). The ABI shares similar spectral sensitivity with imagers on the Japanese Himawari 8/9 mission (Bessho et al. 2016), the Chinese Fengyun‐4 series (Wang et al. 2023), Korean GEO‐KOMPSAT (Kim et al. 2021), and European Meteosat‐12 (Ghilain et al. 2011). A challenge has been integrating geostationary satellites into consistent near‐global coverage, spearheaded by the GeoNEX initiative (Nemani et al. 2020).

In 2018, NASA launched the ECOsystem Spaceborne Thermal Radiometer Experiment on Space Station (ECOSTRESS) mission, which was the first spaceborne mission with a central focus on ET (Fisher et al. 2020). ECOSTRESS was launched to the International Space Station, measuring TIR at 70 m between 52 S—52 N latitudes with varying overpass time of day and revisit periods every few days. ECOSTRESS operationally produced ET data for near‐global coverage using PT‐JPL and for the continental US using ALEXI/DisALEXI, later expanding to include additional models. The increased spatiotemporal resolution afforded by ECOSTRESS has opened analyses into cloudy regions of the world, such as the Tropics, by increasing the probability of seeing between clouds (Doughty et al. 2023).

Still in the decade of the 2010s, a new class of remote sensing‐based ET models emerged, featuring increased complexity and physical realism. Both published in 2011, Breathing Earth System Simulator (BESS) (Ryu et al. 2011) and Global Land Evaporation Amsterdam Model (GLEAM) (Miralles et al. 2011) introduced additional dimensions: for BESS, leaf angle, canopy nitrogen, photosynthesis, and diffuse/direct radiation; for GLEAM, interception evaporation, soil moisture, and vegetation water content (the latter two using microwave data). Additionally, the Penman‐Monteith equation was revisited in four new models: ETLook in 2012 (Bastiaanssen et al. 2012), Surface Temperature Initiated Closure (STIC) in 2014 (Mallick et al. 2014), ETMonitor in 2015 (Hu and Jia 2015), and Penman‐Monteith‐Leuning (PML) in 2016 (Zhang et al. 2016). While ETLook added complexity and partitioning, coupling precipitation to Penman‐Monteith, and ETMonitor included microwave data and expanded complexity into the Shuttleworth and Wallace (1985) framework, STIC and PML simplified Penman‐Monteith linking the surface resistances to TIR (STIC) and LAI (PML). ETLook forms the core of the Water Productivity through Open access of Remotely sensed derived data (WaPOR) dataset from the United Nations Food and Agriculture Organization (UN FAO) (Mannaerts et al. 2020). As with GLEAM, the use of microwave data in ETMonitor enabled retrievals of ET beneath clouds, which is a limitation of TIR/VNIR‐based ET. Further, a new version of PT‐JPL included microwave‐based soil moisture from the Soil Moisture Active‐Passive (SMAP) mission, launched in 2015 (Entekhabi et al. 2010). Both STIC and this new version, PT‐JPLsm (Purdy et al. 2018), were later incorporated into the ECOSTRESS data production. A comprehensive overview summarizing many of these models and their practical functionalities was published by the UN FAO (FAO 2023) relative to the classical Kc ET0 approach (Doorenbos et al. 1986), also covered recently by the UN FAO (Santos Pereira et al. 2025).

## 5 New Players and New Game: ET Everywhere, All the Time

While the US government (NASA, NOAA, USGS) pioneered the foundational satellites of Earth observation for ET (and with continued launches, for example, the next VIIRS instruments launched on NOAA 20/JPSS‐1 and NOAA‐21/JPSS‐2 in 2022), the European Union (ESA) soon joined, followed by new global players. The China National Space Administration (CNSA) has launched numerous satellites with TIR and VNIR imagers, though not necessarily all publicly available or yet broadly used (Chen et al. 2025), across the Ziyuan series (ZY‐3 in 2012 in partnership with Brazil, ZY‐1 02E in 2021), Gaofen series (GF‐4 in 2015, GF‐5 in 2018, GF‐5 01A in 2022), Fengyun series (FY‐3 in 2021, FY‐4 in 2023), and SDGSAT‐1 in 2021 (Guo et al. 2023; Pollpeter 2015; Xian et al. 2021; Yao et al. 2020).

The Indian Space Research Organisation (ISRO) also launched TIR imagers on INSAT‐3D in 2013, INSAT‐3DR in 2016, and EOS‐08 in 2024 primarily for weather monitoring (Gopikrishnan et al. 2023; Kumar et al. 2025). More notably for ET, ISRO, with the French Centre National d'Études Spatiales (CNES), developed the Thermal infrared Imaging Satellite for High‐resolution Natural resource Assessment (TRISHNA) mission, with a launch date in 2026, featuring four TIR spectral bands at 60 m and a three‐day revisit (Lagouarde et al. 2018). TRISHNA marks the first inter‐governmental space agency collaboration for ET, with NASA in partnership with the Italian Space Agency (ASI) planning for a Surface Biology and Geology (SBG) mission (redesigned and named 'Eagle') in the early 2030s (Cawse‐Nicholson et al. 2021; Voosen 2026), ESA developing the Land Surface Temperature Monitoring (LSTM‐A and LSTM‐B) for launches in 2029 and 2031 (Koetz et al. 2018), and NASA planning Landsat Next for launch in 2031 (Roy et al. 2026), all with 50–60 m spatial resolution. The NASA/NOAA GeoXO imagers in development will continue to improve spatial and spectral resolutions beyond the GOES missions planned for 2032, 2034, 2039, and 2043 (Lindsey et al. 2024). None of these missions will themselves provide daily global revisit, but in combination they would approach this critical temporal frequency. TRISHNA has a 5‐year expected lifespan, so any delays from the others puts the combined daily cadence at risk.

It has long been established that daily high‐resolution ET is needed to answer key science questions and societal applications (Cawse‐Nicholson et al. 2021; Fisher et al. 2017; National Academies of Sciences et al. 2019). From assessment of heterogeneous ecosystems and riparian corridors to monitoring of agricultural fields and water management, ET is needed at resolutions that capture the spatial and temporal dynamics of these systems. For example, the average agricultural field for most of the world is < 80 m length scales, necessitating pixel sizes of at most half that to minimize mixed pixel contamination (Lesiv et al. 2019). Temporally, ET can change significantly from day to day as the weather changes, soil moisture depletes past critical thresholds, and irrigation schedules shift water availabilities (Guillevic et al. 2019; Huang et al. 2025). Yet, none of the aforementioned missions achieve such high spatiotemporal resolutions, limiting our ability to have ET everywhere, all the time. This gap may soon be filled with new players and new rules to the game.

**FIGURE 2:**
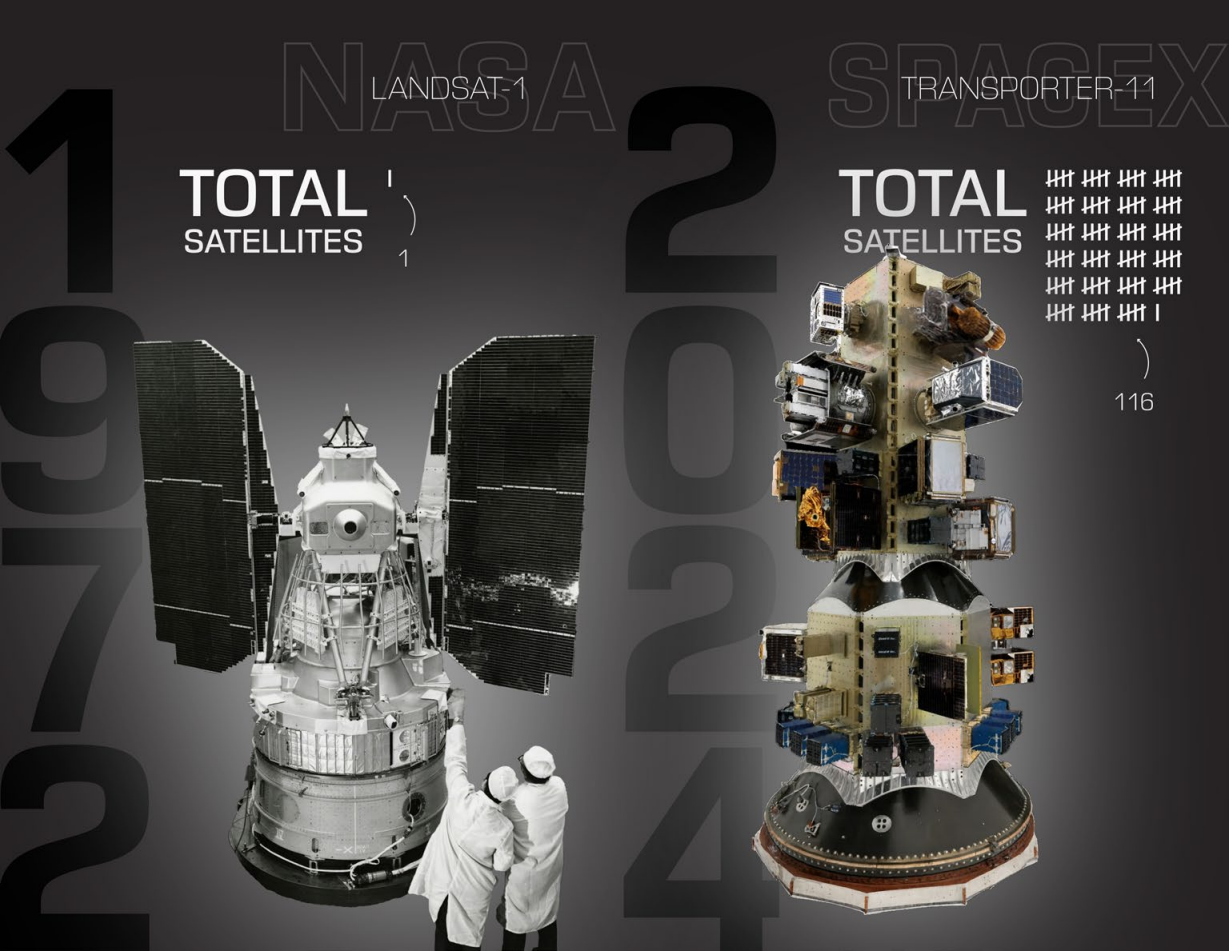
(Left) Landsat‐1 being prepared for launch in 1972 (image credit: NASA). (Right) SpaceX's Transporter‐11, which launched in 2024, carried on it over a hundred smallsats (image credit: SpaceX). Smallsats are being launched with unprecedented frequency and quantity, measuring across the electromagnetic spectrum in increasingly higher spatial resolution, revisit cadence, and spectral resolution.

The newest players in the satellite ET arena come from the commercial space industry, which is launching TIR and VNIR satellites with unprecedented resolutions, frequencies, and launch cadence (Figure 2). Hydrosat, in particular, is leading in this space with VanZyl‐1 launched in 2024 and VanZyl‐2 in 2025, en route to 16 satellites providing TIR at 25 m and VNIR at 10 m globally, multiple times per day (Fisher 2022). Constellr is also developing high‐resolution 30 m TIR microsatellites with SkyBee‐1 and SkyBee‐2 launched in 2025 as part of their High‐precision Versatile Ecosphere (HiVE) constellation (Spengler et al. 2024). EarthDaily will acquire TIR data across 10 satellites at 120 m daily and globally, with their first launch in 2025 and their other satellites forthcoming (Clenet et al. 2023). Other exciting technology comes from SatVu (sponsored in part by the UK Space Agency, UKSA) and Albedo, which have brought spaceborne TIR measurements down to < 5 m, though for very localized applications (Orusa et al. 2024). New low‐latency measurements from FireSat, OroraTech, and WildFireSat (the latter two supported in part by the Canadian Space Agency, CSA) bring complementary measurements in the midwave infrared (MWIR), though these focus on fire detection rather than ET (Honary et al. 2025). Finally, a host of companies now provide ultra‐high resolution VNIR data globally and daily, including Planet (3 m) and Maxar/Vantor (50 cm), from hundreds of satellites in orbit (Bennett et al. 2022). These new smallsat constellations are synergistic with the governmental missions (e.g., Landsat) through calibration and combined data to increase coverage.

Until recently, data acquired by satellites and their corresponding data products were processed and stored on local computers and servers. The new data arena, however, is in the cloud. Services such as Amazon Web Services (AWS) and Google Earth Engine (GEE) now allow the new massive amounts of data to be processed and accessed within minutes, orders of magnitude faster than traditional servers (Amani et al. 2020; Li et al. 2010). Some of the highest quality ET data come from the OpenET system on GEE, which synergizes multiple ET models together into an ensemble product (Melton et al. 2022). The cloud is “everywhere and all the time”, which is a necessary pairing with the new era of TIR and ET data that are very closely approaching the same spatiotemporal ubiquity. Nevertheless, questions about access and cost remain but will be resolved in the years to come.

ET model developments have recently shifted—though derivatives of the aforementioned models continue to propagate (Yao et al. 2026)—toward fusion of process‐based models and satellite/weather data with machine learning (ML) into hybrid models (Guo et al. 2024; Koppa et al. 2022; Miralles et al. 2025; Shang et al. 2021; Xu et al. 2018; Yao et al. 2017). Upscaling eddy flux towers using remote sensing data across different ML models has led to the FLUXCOM and X‐BASE datasets (Jung et al. 2020; Nelson et al. 2024) and the ALIVE system, the latter providing ET in near real‐time over CONUS (Ranjbar et al. 2025). Blending together different process‐based models with a multitude of ML techniques and Bayesian inference has produced the Global Land Surface Satellite (GLASS) dataset (Xie et al. 2022; Yao et al. 2014).

## 6 Conclusion

We showed that remotely sensed ET development is like a river, clear as crystal, proceeding out of decades of technical and scientific progress. From the first NASA/USGS Landsat and development of ET models that leveraged TIR, VNIR, and weather data, through the steady progression of increasingly higher spatiotemporal resolution TIR and VNIR across international space agencies and commercial industry along with increasing sophistication of ET models leveraging cloud computing and machine learning, we have marched forward towards ET everywhere, all the time. Models formerly using high‐resolution data only for local applications have now converged with models that used coarse‐resolution data for global applications through an increase in domain in the former and processing power for the latter, making local relevance now global. For example, data from Landsat, ECOSTRESS, MODIS, VIIRS, Sentinel‐2AB, and Hydrosat are all integrated together into Hydrosat's IrriWatch application delivering data to millions of farmers every day at 10 m resolution in over 70 countries, making a substantial improvement in irrigation and crop management. Like flux towers in the sky, the future of ET is more satellites, higher spatial resolution and frequency, new processing pipelines, and more data with easier access for all, fostering broader applications in sustainable water management and climate resilience (Fisher et al. 2017; Huang et al. 2025; Schimel et al. 2019; Yi et al. 2024).

## Author Contributions

**Joshua B. Fisher:** conceptualization, data curation, formal analysis, funding acquisition, investigation, methodology, project administration, resources, supervision, visualization, writing – original draft, writing – review and editing. **Martha C. Anderson:** writing – original draft, writing – review and editing. **Diego G. Miralles:** writing – original draft, writing – review and editing. **Kanishka Mallick:** writing – original draft, writing – review and editing. **Paul C. Stoy:** writing – original draft, writing – review and editing. **Youngryel Ryu:** writing – original draft, writing – review and editing. **Wim G. M. Bastiaanssen:** writing – original draft, writing – review and editing.

## Funding

J.B.F. was supported in part by NASA ECOSTRESS Science and Applications Team (ESAT) (80NSSC23K0309), NASA Earth Science Applications: Water Resources (WATER) (80NSSC22K0936), and NASA SMD Bridge Program Seed Funding (BPSF) (80NSSC24K1617). D.G.M. was supported by the European Research Council (ERC) via the HEAT Consolidator grant (101088405) and the European Space Agency (ESA) via the CCI Land Evaporation project (4000147355/25/I‐LR). P.C.S. was supported in part by the National Science Foundation Hydrological Sciences award 2422397. K.M. was supported by the HiDRATE project funded by FNR‐ANR Inter programme (Contract No INTER/ANR/22/17204507/HiDRATE), and duly acknowledges CNES/TOSCA funding for TRISHNA Ecosystem Stress project. We acknowledge support from the NSF Division of Earth Sciences (2012893) through CUAHSI and the USGS John Wesley Powell Center for Analysis and Synthesis.

## Conflicts of Interest

The authors declare no conflicts of interest.

## Data Availability

No primary data presented in this paper.
